# Too healthy for their own good: orthorexia nervosa and compulsive exercise in the community

**DOI:** 10.1007/s40519-023-01575-y

**Published:** 2023-06-27

**Authors:** Ada H. Zohar, Merav Zamir, Lilac Lev-Ari, Rachel Bachner-Melman

**Affiliations:** 1 Clinical Psychology Graduate Program, Ruppin Academic Center, Emek Hefer, Israel; 2 Lior Zfaty Suicide and Mental Pain Research Center, Emek Hefer, Israel; 3 School of Social Work, Hebrew University, Jerusalem, Israel

**Keywords:** Orthorexia, Eating disorders, Parental feeding practices, Compulsive exercise, Emotion dysregulation

## Abstract

**Background:**

The similarities and differences between orthorexia nervosa symptoms (ONs) and the symptoms and correlates of eating disorders listed in the DSM-5 need to be elucidated. ONs were examined in a volunteer community sample in conjunction with compulsive exercise, disordered eating, as well as emotional and behavioral correlates of eating disorders.

**Methods:**

Participants were 561 adult volunteers (93 men, 17.09%) aged 19–72 (*M* = 32.7 ± 11), recruited via social media networks. Participants self-reported on the following measures online: Düsseldorf Orthorexia Scale*,* Compulsive Exercise Test, Retrospective Child Feeding Questionnaire, Experiences in Close Relationships, Difficulties in Emotional Regulation Scale, Toronto Alexithymia Scale, and Eating Disorder Examination Questionnaire 13. Data were downloaded and analyzed in SPSS26 and Amos26.

**Results:**

A theoretical model of the connections between the study variables was tested via SEM and confirmed. The profiles of participants with high, average and low levels of ONs were compared. Participants with the highest levels also scored highest for compulsive exercise, insecure attachment, alexithymia, emotion regulation difficulties, weight and shape concerns, body dissatisfaction, restriction, bingeing, purging and parental feeding practices of concern about child’s weight and restricting and monitoring the child’s intake of calorie-rich foods.

**Discussion:**

High levels of ONs are related to disordered eating attitudes and behaviors, as well as to emotional and behavioral correlates of eating disorders. It is unclear to what extent these ONs are distinguishable from symptoms of other eating disorders listed in the DSM-5. Longitudinal studies may help to elucidate distinct trajectories and risk factors for ON.

*Level of evidence*: Level III, case–control analytic study.

## Introduction

The focus of this study is orthorexia nervosa (ON), or strict adherence to and preoccupation with healthy eating, emotional distress from failure to adhere to this regimen, and a resulting restriction of social intercourse or other functional impairment [1]. In their proposed criteria for ON, Dunn and Bratman [2] specify in criterion A (core impairment), that the obsessive focus on health might lead to weight loss but is not focused on weight loss as in anorexia nervosa (AN). They also specify that the dietary restrictions in ON are characterised by a spiralling course that can lead to the exclusion of whole food groups, as well as repeated fasts intended to cleanse or purify. In their narrative review, Cena et al., [3] point out that the following advectives are commonly mentioned in descriptions of ON: “obsessive, exaggerated/excessive, unhealthy, compulsive, pathological, rigid/controlling, extreme, monoideistic, maniacal, time-consuming, and overwhelming”. Donini et al. [4] further specify that in ON, the obsession with food intake results in very detailed planning of one’s daily routine and many hours per day are devoted to mapping out activities. As a consequence, plans become inflexible and minor changes to them can cause great distress.

ON is associated with health behaviors, such as physical exercise. Bóna et al. [5] found elevated ONs among Hungarian gym members, which were positively associated with drive for thinness and interpersonal distrust. Physical exercise to avoid negative emotions linked to weight and shape concerns verges on compulsive exercise and is found also in elite athletes [6]. Strahler et al. [7] found the association between ON and compulsive exercise to be moderate in women and stronger in men; in women these variables were related to trait anxiety. Compulsive exercise and ON are seemingly healthy behaviors but excessive and potentially injurious, crossing the line into ill-health and dysfunction [8]. ON and compulsive exercise share a rigid and extreme nature that can synergistically cause physical problems and injury as well as emotional distress. In this study we consider the possibility that an obsessive emphasis on health can drive both ONs and compulsive exercise, and we relate to both as outcomes. There may well be a positive feedback cycle so the ONs feed compulsive exercise, which in turn feeds ONs.

There are well-established risk factors for EDs which might be important in the context of ON and ONs. Insecure attachment was found to be associated with eating disorders and disordered eating [9, 10]. Emotional regulation difficulties and alexithymia also characterize people with eating disorders and seem to improve with recovery [11]. In an unpublished thesis, Muno [12] reported significant correlations between levels of ONs, alexithymia and insecure attachment in college students. Vuillier et al. [13] found positive correlations between ONs and disordered eating, difficulty in identifying one’s emotions and emotion dysregulation. Obeid et al. [14] found a positive and significant correlation between orthorexic symptoms, alexithymia, and difficulty in emotion regulation in a large population sample. Previous research, therefore, suggests that initial emotional insecurity, difficulty in identifying and describing one’s emotions, and difficulty in regulating emotions may be elevated in individuals high in ONs, just as they are in people with symptoms of eating disorders. Clearly, emotional (dys)function in the context of ONs requires further research.

There are behavioral experiential risk factors for eating disorders which may also play a role in ON and ONs. Much evidence links early parental feeding practices to disordered eating [15] and eating disorders [16]. Recalled parental child feeding practices are associated, in adults, with disordered eating, BMI, and body dissatisfaction [17, 18]. However, the association between parental feeding practices and ONs has not yet been examined.

**Fig. 1:**
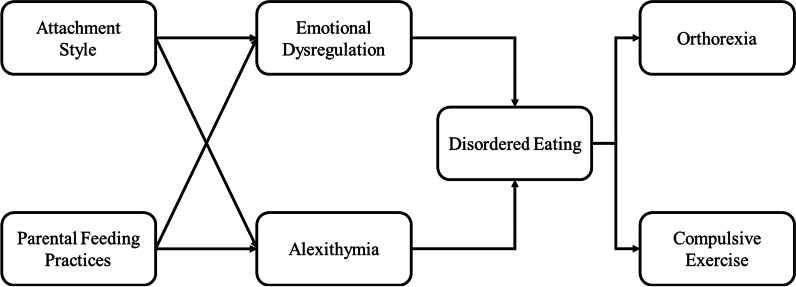
Hypothesized model of connections

The theoretical model we test in this study follows a developmental logic, considering attachment style and parental feeding practices, which might in turn affect emotional function and disordered eating. Emotional function and disordered eating may serve as mediators between these early influences and the two main study outcomes—ONs and compulsive exercise. Early distress in infants can be relieved by sensitive and responsive parenting, which includes feeding the infant when hungry and responding to the other needs of the infant differently, without offering food in response to distress [19]. This helps infants to recognize hunger and satiety cues, and later helps the developing child to develop secure attachment, recognize their emotions and differentiate them from hunger. Parental feeding practices that are not responsive to the child’s needs are associated in young children with insecure attachment, problematic eating behaviors, and emotional difficulties [20, 21]. An association between parental feeding practices, insecure attachment and eating pathology has been observed in early adolescence as well [22]. Insecure attachment and maladaptive feeding practices can lead, in conjunction with environmental influences on health beliefs, to disordered eating and emotional dysfunction. These influences may feed into ONs and compulsive exercise that can then work synergistically to exacerbate each other and blossom into full blown ON. This theoretical model is presented in Fig. 1.

## Methods

### Participants

Our initial power analysis, conducted in GPower [23] for analysis of variance comparing 3 groups on up to 10 variables with an *α* of 0.05 yielded 95% power for a sample of 522. Thus, recruitment continued until and slightly after that number of participants had sent in complete responses. Participants were 561 adult volunteers aged 19 to 72 years (M = 32.7 ± 11), recruited via social media networks, facebook pages and whatsapp groups. The sample was mostly female, with 93 (17.09%) men. Three participants did not report gender. A minority (n = 115, 19.9%) had up to 12 years of education, 255 (45.4%) were undergraduates, 156 (27.76%) had an MA and 35 (6.23%) a PhD. Participants’ self-reported BMI ranged from 15 to 55 (M = 23.58 ± 4.49). 4.5% reported a BMI of up to 18, 65% from 18 to 25, 20% from 25 to 30 and 10% over 30.

### Measures

Retrospective recall of parental feeding practices during childhood were assessed using the 28-item Retrospective Child Feeding Questionnaire (RCFQ) [17]. The RCFQ consists of six subscales: (1) Perceived Mother's Weight (PMW); (2) Perceived Child's Weight (PCW); (3) Concern about the Child being Overweight (CCO); (4) Restriction (R); (5) Pressure to Eat (PE); and (6) Monitoring (M). Participants rate their retrospective childhood memories, i.e., to what extent their mother was concerned about their weight and restricted, pressured and monitored their eating. Responses are rated on a scale of 1–5. The RCFQ has shown convergent validity with adults’ BMI, body dissatisfaction [17] and emotional and non-intuitive eating in young adults [24]. The subscales have good internal consistency, with Cronbach's alpha between 0.73 and 0.91 [17, 25]. In this study Cronbach's alphas were *α* = 0.79 (PMW), *α* = 0.80 (PCW), *α* = 0.84 (CCO), *α* = 0.80 (R), *α* = 0.73 (PE) and *α* = 0.92 (M).

Attachment styles were assessed via the 12-item Experiences in Close Relationships-12 (ECR-12) [26]. Two dimensions of attachment styles are included: Anxiety and Avoidance of Close Relationships, with items rated between 1 and 7. Lafontaine et al. [26] found the psychometric properties of the ECR-12 to be as sound as those of the 36-item version [27]. The ECR-12 has good internal consistency, with Cronbach's alpha of the Hebrew version ranging from 0.88 to 0.91 [28]. In this study, Cronbach's alphas were *α* = 0.84 (avoidant) and *α* = 0.87 (anxious).

Emotional dysregulation was measured using the 36-item Difficulties in Emotion Regulation Scale (DERS) [29], across six subscales: (1) Non-acceptance; (2) Goals; (3) Impulse; (4) Lack of awareness; (5) Strategies; and (6) Clarity. Responses are rated on a scale of 1–5 and the sum of the subscale scores forms a global score. The DERS ( Hebrew version has good validity and reliability [29, 30]. In this study Cronbach's alpha was *α* = 0.95.

Alexithymia was assessed with the 20-item Toronto Alexithymia Scale 20 (TAS-20) [31]. The TAS-20 evaluates three dimensions of alexithymia: Difficulty Identifying Feelings, Difficulty Describing Feelings and Externally Oriented Thinking. Items are rated on a scale of 1–5 and the sum of the subscales form a global score. The TAS-20 has good internal consistency, test–retest reliability, and convergent, discriminant and concurrent validity [31]. The Hebrew version also has good internal reliability. α = 0.88; [32]. In this study Cronbach's alphas were *α* = 0.85 (Difficulty Identifying Feelings), *α* = 0.78 (Difficulty Describing Feelings), *α* = 0.68 (Externally Oriented Thinking) and *α* = 0.86 (Total).

Disordered eating was measured by the 13‐item Eating Disorder Examination—Questionnaire-13 (EDE-Q-13); [33]. The EDE‐Q-13 is a short version of the 28‐item EDE‐Q [34], which was translated into Hebrew and validated [35]. The EDE-Q-13 is composed of five subscales: Shape and Weight Over-evaluation, Eating Restraint, Body Dissatisfaction, Bingeing and Purging. Items are rated on a scale between 0 and 6 and the sum of the subscale scores forms a global score. The EDE‐Q-13 has good construct validity and internal consistency, with Cronbach's alpha of subscales between 0.63 and 0.99 [33]. In this study Cronbach's alpha was *α* = 0.89.*α* = 0.79

Orthorexic eating behaviors and associated emotions were assessed using the 10-item Düsseldorf Orthorexia Scale (DOS) [36]. Each item is rated on a scale of 1–4 and the clinical cutoff point is 30, with participants scoring 25–29 considered at risk for ON. The English translation of the original German has good psychometric properties [1]. The English version was translated into Hebrew for this study, with permission from the authors, via translation and independent back-translation by two bilingual translators, who resolved discrepancies and agreed on the final wording. In this study Cronbach's alpha was *α* = 0.83.

Level of compulsive exercise was assessed by the 24-item Compulsive Exercise Test (CET) [8]. The CET is comprised of five subscales: (1) Avoidance and Rule-Driven Behavior; (2) Weight Control Exercise; (3) Mood Improvement; (4) Lack of Exercise Enjoyment; and (5) Exercise Rigidity. Each item is rated on a scale of 0–5. The CET has good internal consistency, with Cronbach's alpha between 0.85 and 0.88 [40.6]. The questionnaire was translated into Hebrew, with permission from the authors, similar to the DOS. In this study Cronbach's alpha was *α* = 0.93.

### Procedure

The study was approved by the Institutional Review Board. Participants were sent a link to the questionnaires via Qualtrics (www.qualtrics.com). On the opening screen, potential participants were given information about the study’s aims and content, and were informed they could stop participating at any point. Those participants who subsequently provided informed consent and completed the measures described above were included in this study. No reward was offered for participation. APA ethical guidelines were adhered to throughout.

### Data analyses

Associations between quantitative variables were assessed using Pearson correlations and associations between qualitative variables using Chi-Square. A structural equation model (SEM) tested our hypothesized model. SEM was chosen because of its suitability for testing a complex theoretical model, and because we expected shared variance between most of the study variables, which SEM is designed to take into account. SEM also has the advantage of being both confirmatory and exploratory; it goes beyond the theoretical model by testing the strength of all potential paths between the model variables [37]. Analysis of variance with Bonferroni range-correction for significance level for post-hoc comparisons was used to compare the three levels of ON produced by DOS scores. Statistical Package for the Social Sciences (SPSS, version 26) and AMOS 26 were used. Analyses were tested for statistical significance at the *p* < 0.05 level.

## Results

### Descriptive statistics

Means and standard deviations for study variables are shown in Table 1.

**Table 1:** Means and standard deviations of study variables

	*M*	SD
*RCFQ*		
Perceived mother's weight	2.64	0.68
Perceived child's weight	2.66	0.61
Concern about child’s overweight	1.82	0.99
Restriction	2.07	0.79
Pressure to eat	2.80	1.06
Monitoring	2.33	1.05
*ECR-12*		
Avoidant	3.61	1.27
Anxiety	3.62	1.45
*DERS*	2.28	0.64
*TAS-20*	2.20	0.54
Difficulty identifying feelings	2.28	0.78
Difficulty describing feelings	2.33	0.81
Externally oriented thinking	2.05	0.52
Total		
*EDE-Q-13*		
Shape and weight over-evaluation	3.74	2.30
Eating restraint	3.33	2.12
Body dissatisfaction	3.52	2.14
Bingeing	1.85	1.24
Purging	1.11	0.43
Total	2.58	1.21
*DOS*	17.0	5.20
*CET*	3.22	1.01

*RCFQ*  Retrospective Child Feeding Questionnaire, *ECR-12*  Experiences in Close Relationships-12, *DERS*  Difficulties in Emotion Regulation Scale, *TAS-20*  Toronto Alexithymia Scale-20, *EDE-Q-13*  Eating Disorder Examination Questionnaire-13, *DOS*  Düsseldorf Orthorexia Scale, *CET*  Compulsive Exercise Test

There was a strong non-parametric association between DOS and the CET, with 92.3% of the participants scoring above the DOS cutoff point for clinical ON scoring above the CET median *Χ*^2^ = 24.3, *p* < 0.0001).

Bivariate associations between the study variables (Pearson correlations) are shown in Table 2.

**Table 2:** Pearson correlation coefficients between study variables

	1	2	3	4	5	6	7	8	9	10	11	12	13
1. RCFQ-PMW	–	0.26***	0.07	0.08*	− 0.02	0.05	0.06	0.09*	0.11**	0.06	0.15***	0.09*	0.07
2. RCFQ-PCW		–	0.33***	0.30***	− 0.09*	0.23***	0.10*	0.10*	0.12**	0.12**	0.31***	0.13**	0.12**
3. RCFQ-CCO			–	0.59***	0.13**	0.48***	0.04	0.24***	0.16***	0.13**	0.33***	0.26***	0.14***
4. RCFQ-R				–	0.23***	0.66***	0.09*	0.33***	0.29***	0.26***	0.30***	0.20***	0.16***
5. RCFQ-PE					–	0.14***	0.10*	0.16***	0.13**	0.13**	− 0.01	0.05	0.01
6. RCFQ-M						–	0.04	0.27***	0.21***	0.17***	0.31***	0.27***	0.23***
7. ECR-12-avoidant							–	0.14***	0.25***	0.45***	0.10*	0.08	− 0.05
8. ECR-12-anxiety								–	0.58***	0.38***	0.28***	0.12**	0.13**
9. DERS									–	0.66***	0.37***	0.16***	0.13**
10. TAS-20										–	0.28***	0.14***	0.10*
11. EDE-Q-13											–	0.52***	0.37***
12. DOS												–	0.53***
13. CET													–

*RCFQ-PMW*  Retrospective Child Feeding Questionnaire-Perceived Mother's Weight, *RCFQ-PCW*  Retrospective Child Feeding Questionnaire-Perceived Child’s Weight, *RCFQ-CCO*  Retrospective Child Feeding Questionnaire-Concern about Child’s Overweight, *RCFQ-R*  Retrospective Child Feeding Questionnaire-Restriction, *RCFQ-PE*  Retrospective Child Feeding Questionnaire-Pressure to Eat, *RCFQ-M*  Retrospective Child Feeding Questionnaire-Monitoring, *ECR-12*  Experiences in Close Relationships-12, *DERS* Difficulties in Emotion Regulation Scale, *TAS-20*  Toronto Alexithymia Scale-20, *EDE-Q-13*  Eating Disorder Examination Questionnaire-13, *DOS* Düsseldorf Orthorexia Scale, *CET*  Compulsive Exercise Test

^*^p < 0.05; ** *p* < 0.01; *** *p* < 0.001

To test the hypothesized theoretical model, we used structural equation modelling (SEM) via AMOS 26. As a combined rule for the acceptance of our model, we chose the following values: Normed Fit Index (NFI) > 0.90 [38, 39] and Root Mean Square Error of Approximation (RMSEA) < 0.08 [39]. The Chi Square goodness-of-fit index presented an excellent fit for the data, χ^2^_(16)_ = 11.438, *p* = 0.782; NFI = 0.993; CFI = 1.000; RMSEA = 0.000.

Overall, the hypothesized model was confirmed. Avoidant attachment had a positive path to emotional dysregulation and alexithymia, and a negative path to compulsive exercise. Anxious attachment and parental feeding restriction were positively related to emotional dysregulation and alexithymia. Parental feeding monitoring was directly and positively associated with disordered eating, ONs and compulsive exercise. Emotional dysregulation was positively associated with disordered eating, which was in turn positively associated with ONs and compulsive exercise. Contrary to our prediction, alexithymia was not associated with disordered eating, ONs or compulsive exercise.

**Fig. 2:**
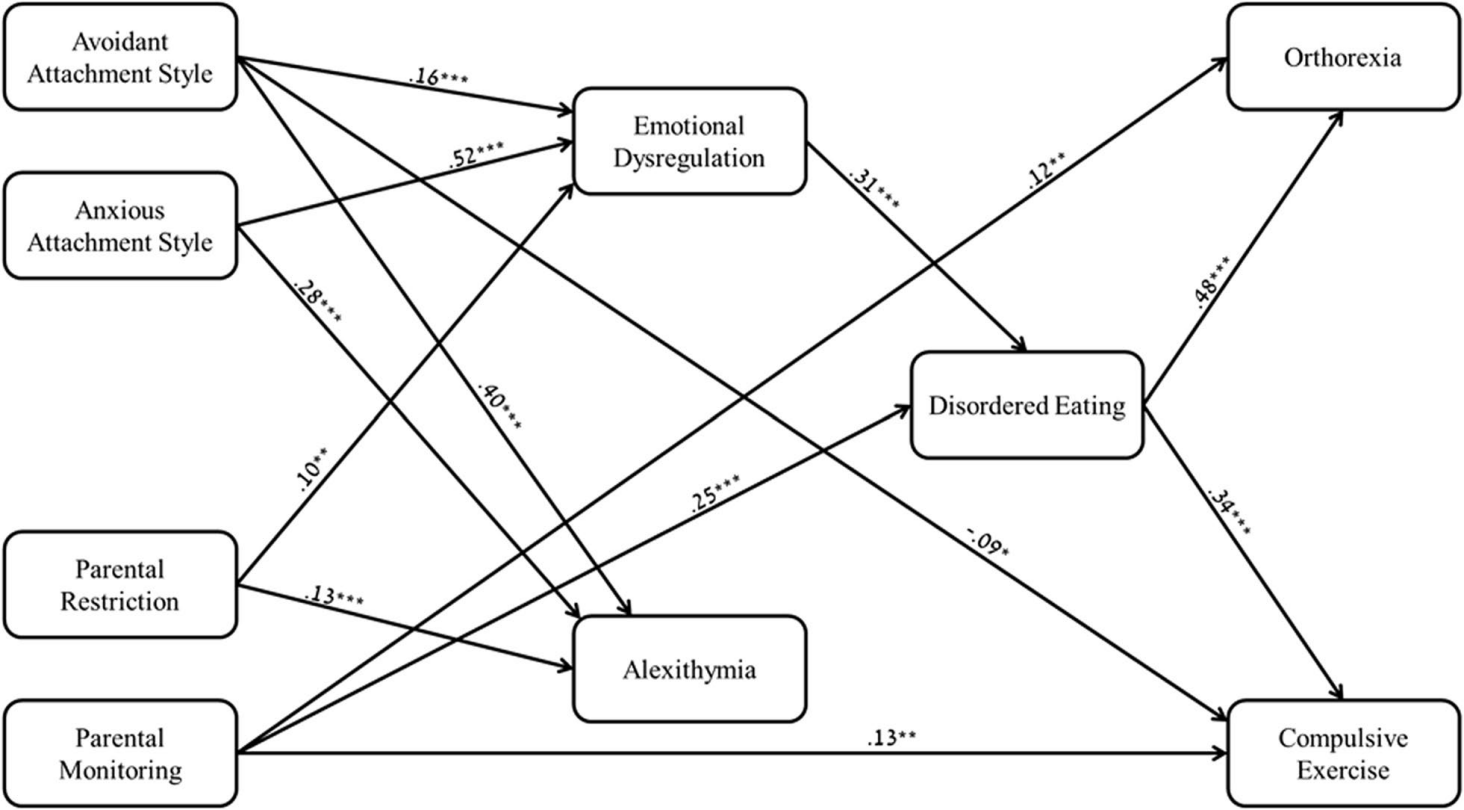
SEM model: paths between study variables. *p < 0.05; **p < 0.01; ***p < 0.001

Parental restriction and both avoidant and anxious attachment were indirectly associated with ONs and compulsive exercise via emotional dysregulation, which was associated with disordered eating. Parental monitoring was indirectly associated with ONs and compulsive exercise via disordered eating (see Fig. 2).

As a further test of the relationship between orthorexic symptoms and other study variables, we compared the three levels of orthorexic symptoms using analysis of variance, as suggested by [40]. DOS scores ranged from 10 to 38 (potential range 10–40, mean = 17 ± 5.2. Only 15 (2.6%) of the participants scored over the DOS cutoff of 30. At-risk individuals scoring 25–29 comprised 4.9% of the sample (*N* = 27), and the remainder (*N* = 519, 92.5%) scored under 25. The ANOVA command in SPSS was used with Bonferroni range correction for post-hoc between-group comparisons.

Those with DOS scores above 30 recalled the most maternal concern over their (over)weight, and maternal restriction and monitoring of their calorie-rich snacks as children. Post-hoc comparisons showed significant differences between participants with high (> 30) and low (< 25) DOS scores. Those scoring above 30 scored highest for anxious attachment, emotional dysregulation, alexithymia, weight and shape concerns, body dissatisfaction, restriction, purging, bingeing, and compulsive exercise (see Table 3).

**Table 3:** Analysis of variance for study variables for the three DOS subgroups

Variable	Low DOS level	At-risk DOS level	Clinical DOS level	*F*(*p*)
*RCFQ*				
Perceived mother's weight	2.64 (0.67)	2.58 (0.72)	2.90 (0.64)	1.02 (0.36)
Perceived child's weight	2.65 (0.61)	2.73 (0.64)	2.85 (0.55)	0.96 (0.38)
Concern about child’s overweight^a,b^	1.78 (0.96)	2.21 (1.32)	2.50 (0.86)	6.26 (< 0.01)
Restriction^b^	2.04 (0.76)	2.35 (1.08)	2.87 (0.68)	8.98 (< 0.001)
Pressure to eat	2.79 (1.04)	3.17 (1.23)	2.77 (0.87)	2.14 (0.12)
Monitoring^a,b,c^	2.26 (0.99)	2.81 (1.46)	3.67 (0.70)	15.31 (< 0.001)
*ECR-12*				
*DERS*	2.25 (0.62)	2.44 (0.72)	2.70 (0.89)	4.15 (0.02)
*TAS-20*	2.18 (0.54)	2.36 (0.59)	2.53 (0.63)	4.17 (0.02)
*EDE-Q-13*				
Shape and weight over-evaluation^a,b,^	3.54 (2.25)	5.65 (1.87)	6.67 (0.62)	26.28 (< 0.001)
Eating restraint^a,b^	3.13 (2.03)	5.22 (2.05)	6.25 (1.10)	30.96 (< 0.001)
Body dissatisfaction^a,b^	3.38 (2.09)	4.92 (2.16)	5.21 (2.12)	13.03 (< 0.001)
Bingeing^a,b^	1.79 (1.19)	2.41 (1.48)	2.81 (1.87)	8.10 (< 0.001)
Purging^a,b,c^	1.07 (0.34)	1.33 (0.65)	2.17 (1.19)	50.18 (< 0.001)
*CET*^a,b^	3.11 (0.95)	4.21 (0.82)	4.64 (0.97)	35.96 (< 0.001)

*RCFQ*  Retrospective Child Feeding Questionnaire, *ECR-12*  Experiences in Close Relationships-12, *DERS*  Difficulties in Emotion Regulation Scale, *TAS-20*  Toronto Alexithymia Scale-20, *EDE-Q-13*  Eating Disorder Examination Questionnaire-13, *DOS* Düsseldorf Orthorexia Scale, *CET* Compulsive Exercise Test

^a^Low DOS Level significantly different from At-Risk DOS Level

^b^Low DOS Level significantly different from Clinical DOS Level

^c^At-Risk DOS Level significantly different from Clinical DOS Level

## Discussion

Findings of this study support the conceptualization of orthorexic symptoms (ONs) within the spectrum of disordered eating attitudes and behaviors. ONs were significantly correlated with disordered eating as measured by the EDE-Q-13, which supports previous findings [40]. They were also associated with insecure attachment, emotional dysregulation and alexithymia, emotional correlates of disordered eating. ONs were also associated with retrospectively reported childhood memories of oneself and one’s mother being overweight, of maternal restriction and monitoring of one’s food consumption, and of maternal concern about the child being overweight. These factors may be precursors of disordered eating and even eating disorders [17, 18]. The distinction between the profiles of participants likely to have ON (DOS scores > 30) and those less at risk was quite dramatic, although only a small number of participants in this study scored over the clinical cutoff point. Between-group differences were significant for all study variables, including all five disordered eating subscales.

Our results also support a close connection between orthorexic symptoms and compulsive exercise. These variables were highly correlated, and both were associated in our SEM model with disordered eating, and with emotional and behavioral factors that contribute to risk for eating disorders. Participants who scored above the DOS clinical cutoff reported significantly higher levels of compulsive exercise than those who did not. A link between ONs and compulsive exercise has been observed in previous research. Oberle et al. [41] reported that students with high levels of ONs had high levels of physical activity and exercise addiction, including intensive exercise despite injury or illness that could lead to physical impairment. In their consensus statement on risk factors for ON, Donini et al., [4] agree that “competitive sports, athletic performance concerns, and high physical exercise frequency” are risk factors for ON.

Much research has focused on restriction as the cognitive and behavioral basis for ON, necessitating differentiation from AN and ARFID. However, in this study, we found that higher levels of ON symptoms are significantly related not only to restriction, but also to bingeing and purging. The questionnaire we used to assess orthorexic symptoms did not ask about these behaviors, but correlations with the bingeing and purging subscales of our measure of disordered eating and comparisons of bingeing and purging behaviors between groups with high, intermediate and low levels of orthorexic symptoms indicated that these symptoms were all significantly linked. Significant, small to moderate associations have in fact frequently been observed between measures of ON and measures of bulimia nervosa symptoms [1, 42–48] as well as with measures of binge eating symptoms that did not assess purging [49].

Our findings, therefore, point to the likelihood that ONs may be better conceived in the realm of eating pathology characterized, inter alia, by bulimic symptoms—binging and purging. It seems that at high levels of ON pathology, symptoms are intertwined with disordered eating of all kinds. Symptoms of all eating disorders, including bulimia nervosa and binge eating disorder often begin with dietary changes motivated by a desire to lose weight or to eat in a healthier way [49, 50]. Our findings support the likelihood that paradoxically, striving for health can lead inter alia, via frustrated dieting, to symptoms of overeating and binge eating. It could also be, that the individuals who scored above the clinical cutoff in the current study had coexisting eating disorders, were masking an eating disorder with health-related cognitions, or were trying to overcome an eating disorder such as AN by turning their perfectionism and obsessiveness from weight and appearance to health concerns [4]. The relationship between ONs and the entire range of eating disorder symptoms clearly need further investigation and clarification.

ON is ego-syntonic, as reflected in DOS item 5: “*I like* that I pay more attention to healthy nutrition than other people”. Individuals contending with ONs, like those with AN symptoms are, therefore, likely to avoid therapy and present for treatment only when symptoms are already severe and extreme. It may then be unclear how distinct their symptoms are from those of another eating disorder. In any case, the emotional correlates of ONs that we found in this study, namely, insecure attachment, emotional dysregulation and alexithymia, would be beneficial to address as needed in relation to an unhealthy obsession with healthy eating. Similar, i.e., insecure attachment styles have previously been observed in ON and clinical eating disorders [51] and a significant and positive association found between ONs and insecure attachment in a non-clinical sample [52]. Our results regarding alexithymia and emotional dysregulation also replicate previous findings [12, 13], so that this study further confirms that ON symptoms are in fact anything but healthy. Individuals with these tendencies can benefit from interventions helping them to identify, express and regulate their emotions. However, the finding that symptoms of ON were significantly linked to maternal restriction and monitoring of the child’s food consumption and maternal concern about her child being overweight is, to the best of our knowledge, a novel finding requiring replication. Interventions targeting these parental feeding attitudes may help protect children from developing an unhealthy obsession with healthy eating. Helping adults to become aware of these issues in the parenting they received may help them overcome current destructive struggles with excessively healthy eating.

### Strengths and limits

The strengths of this study include the large community sample, the use of well-validated measures, and the combination of variables studied in the context of ON, all contributing explained variance. The weaknesses include reliance on self-report alone and the under-representation of men, since ON may well differ between males and females [7], as well as the reliance on a convenience sample rather than a representative population sample. We assessed orthorexic symptoms using the DOS [1], which includes only three ON criteria—rigid dietary regimen, emotional distress caused by violating this regimen, and dysfunction arising from adhering to it. It would have been interesting to include questions about duration of ONs [4], the time involved in planning one’s daily eating schedule, and the course of symptom progression [2]. Since the percentage of participants scoring above the suggested DOS cutoff was low, sampling from health clubs, for example, might have enriched the sample with individuals scoring above the clinical cutoff point [5, 7]. The study was cross-sectional, with all data collected at one single timepoint, limiting our ability to reach conclusions about risk factors and the chronological emergence of the characteristics studied.

### What is already known on this subject

ONs are associated with physical activity, dysfunction and distress. Although there is growing consensus that ON is an eating disorder, its distinction from other eating disorders remains unclear, as do recommended diagnostic criteria.

### What this study adds

The current study supports defining ON as an eating disorder, with bingeing and purging symptoms, weight and shape concerns, as well as restriction. Longitudinal studies should track the development of ON symptoms over time and identify trajectories as well as unique risk and protective factors. Identifying characteristic trajectories would also strengthen the argument for including ON as a Feeding and Eating Disorder in future psychiatric diagnostic systems.

## Data Availability

Data is avilable on request from the corresponding author.
